# 2318. Utility of Routine Asymptomatic Post-Admission SARS-CoV-2 Screening in a Rehabilitation Facility

**DOI:** 10.1093/ofid/ofad500.1940

**Published:** 2023-11-27

**Authors:** Victoria R Williams, Larry Robinson, Morty Eisenberg, Robert Kozak, Kuldeep Virdi, Heather Candon, Jerome A Leis

**Affiliations:** Sunnybrook Health Sciences Centre, Toronto, Ontario, Canada; Sunnybrook Health Sciences Centre, Toronto, Ontario, Canada; Sunnybrook Health Sciences Centre, Toronto, Ontario, Canada; Sunnybrook Health Sciences Centre, Toronto, Ontario, Canada; Sunnybrook Health Sciences Centre, Toronto, Ontario, Canada; Sunnybrook Health Sciences Centre, Toronto, Ontario, Canada; Sunnybrook Health Sciences Centre, Toronto, Ontario, Canada

## Abstract

**Background:**

SARS-CoV-2 testing is recommended for symptomatic patients, and asymptomatic testing following known exposure or on admission during periods of high community incidence. The role of post-admission asymptomatic screening is less clear. We evaluated the impact of routine weekly asymptomatic testing in a 178 bed rehabilitation setting that was experiencing frequent COVID-19 outbreaks following the emergence of the Omicron variant.

**Methods:**

At baseline (April 1, 2021 to March 31, 2022), all patients were tested for SARS-CoV-2 in 4 situations: 1) routinely on admission (≤5 days); 2) in response to the development of new symptoms consistent with COVID-19 infection; 3) following exposure to a patient with COVID-19; and 4) as part of unit wide prevalence testing during suspected or confirmed outbreak. Routine weekly unit wide asymptomatic prevalence screening was introduced April 1, 2022 to March 31, 2023. An uncontrolled before-after study was performed assessing yield of these testing strategies and the incidence of healthcare associated-COVID-19 (HA-COVID). A case was defined as a patient testing positive for SARS-CoV-2 who had not been previously infected with COVID-19 within the preceding 90 days. Patients deemed to be recovered positive based on prospective assessment were excluded.

**Results:**

The intervention was associated with nearly double the number of SARS-CoV-2 tests (8857 vs. 4672), of which 8513 (96.1%) and 4582 (98.1%) were eligible for inclusion during intervention and baseline, respectively. The before-after comparison of testing positivity was similar for overall, admission, and suspect/confirmed outbreaks with yield of 1.4%/0.5%/3.2% versus 2.6%/1.8%/4.9%, respectively. The addition of routine weekly testing had extremely low yield of 0.2% (8/4022). The incidence of HA-COVID increased during the intervention period (3.4 vs. 1.1 per 1000 patient-days; p< 0.001).

**Conclusion:**

In a rehabilitation setting, the addition of routine weekly asymptomatic prevalence testing of patients was of low yield when added to surveillance in place on admission, and for symptomatic or exposed patients. Despite the increased risk of HA-COVID associated with the emergence of the Omicron variant, these findings do not support routine weekly post-admission testing.

**Disclosures:**

**Jerome A. Leis, MD MSc FRCPC**, Ontario Hospital Association, Ministry of Attorney General of Ontario, Seneca College: Expert Testimony

